# Fibronectin matrix-mediated cohesion suppresses invasion of prostate cancer cells

**DOI:** 10.1186/1471-2407-12-94

**Published:** 2012-03-20

**Authors:** Dongxuan Jia, Ildiko Entersz, Christine Butler, Ramsey A Foty

**Affiliations:** 1 Department of Surgery, UMDNJ-Robert Wood Johnson Medical School, 125 Paterson Street, CAB 7319, New Brunswick NJ 08901, USA

**Keywords:** Fibronectin matrix assembly, Tissue surface tension, Tumor cohesion, Invasion suppression, α5β1 integrin

## Abstract

**Background:**

Invasion is an important early step in the metastatic cascade and is the primary cause of death of prostate cancer patients. In order to invade, cells must detach from the primary tumor. Cell-cell and cell-ECM interactions are important regulators of cohesion - a property previously demonstrated to mediate cell detachment and invasion. The studies reported here propose a novel role for α5β1 integrin - the principle mediator of fibronectin matrix assembly (FNMA) - as an invasion suppressor of prostate cancer cells.

**Methods:**

Using a combination of biophysical and cell biological methods, and well-characterized prostate cancer cell lines of varying invasiveness, we explore the relationship between cohesion, invasiveness, and FNMA.

**Results:**

We show that cohesion is inversely proportional to invasive capacity. We also show that more invasive cells express lower levels of α5β1 integrin and lack the capacity for FNMA. Cells were generated to over-express either wild-type α5 integrin or an integrin in which the cytoplasmic domain of α5 was replaced with that of α2. The α2 construct does not promote FNMA. We show that only wild-type α5 integrin promotes aggregate compaction, increases cohesion, and reduces invasion of the more aggressive cells, and that these effects can be blocked by the 70-kDa fibronectin fragment.

**Conclusions:**

We propose that restoring capacity for FNMA in deficient cells can increase tumor intercellular cohesion to a point that significantly reduces cell detachment and subsequent invasion. In prostate cancer, this could be of therapeutic benefit by blocking an early key step in the metastatic cascade.

## Background

Invasion is a critical step in the progression of prostate cancer from a manageable to an intractable disease. In order to invade, tumor cells must detach from the tumor mass. It is widely held that the transition to invasion involves changes in the expression of key cell-cell and cell-ECM adhesion molecules and that these changes facilitate escape of tumor cells and their subsequent spread to other organs in the body. These changes can also signal shifts in key mechanical properties of the tumor. One such property, tumor cohesion, has been demonstrated to influence tumor cell detachment [1-3], and invasiveness of lung [4], muscle [2], and brain [5] tumors. E-cadherin is the predominant cadherin that mediates direct cell-cell cohesion in epithelial tissues. E-cadherin expression in human prostate cancer specimens is significantly down-regulated or absent in high-grade lesions [6]. Interestingly, absence of E-cadherin expression is often associated with an up-regulation of mesenchymal cadherins, including N-cadherin and cadherin-11 [7]. In such cases, net cadherin expression may not necessarily change. Consequently, the overall cohesion of the tumor may be unaffected. Changes in the expression levels of other adhesion systems may also be required to reduce overall tumor cohesion.

Integrin-ECM interactions play a key role in cell adhesion. In prostate cancer, a switch in both integrin expression and in the secretion of an aberrant ECM are associated with progression to invasion [8]. Several studies have reported deregulation of both α and β subunits as prostate cancer progresses [9,10]. Among the α subunits, α5 integrin is down-regulated in adenocarcinoma of the prostate [11]. Alpha - 5 integrin interacts with the β1 subunit to form α5β1 integrin, the primary receptor for fibronectin [12]. Apart from a signaling role in mediating cell proliferation, migration, and differentiation, the interaction between α5β1 integrin and fibronectin promotes the polymerization and assembly of fibronectin into a matrix [13]. Fibronectin polymerization is a critical regulator of extracellular matrix organization and stability [14].

Fibronectin matrix assembly (FNMA) has been shown to markedly influence tissue cohesion [15,16] and to specify liquid to solid phase transitions of 3D cellular [17]. Accordingly, down-regulation of α5 integrin by high-grade prostate cancer cells can, in principle, disrupt matrix assembly, reduce cohesion, and facilitate the detachment of tumor cells from the mass. This was recently reported for a series of glioblastoma (GBM)-derived cell lines. Despite identical pathologic grade, aggregates from these lines dispersed at markedly different rates and dispersal was inversely proportional to capacity for FNMA. Moreover, restoring FNMA in GBM cells markedly reduced their ability to disperse by increasing aggregate cohesion [3]. Little is known regarding the role of fibronectin matrix assembly in mediating prostate cancer cell invasion. An earlier immunohistochemical study showed that in the normal prostate gland, fibronectin expression was restricted to the stromal compartment, whereas α5β1 integrin was predominantly expressed by epithelial cells. In high-grade prostate cancer the expression pattern of fibronectin was patchy and significantly reduced, suggesting either down-regulation of fibronectin secretion or lack of significant organization into a matrix. The study also explored fibronectin secretion by LNCaP cells. Interestingly, incubation of LNCaP cells with an anti-fibronectin antibody resulted in decreased substrate adhesion, suggesting a functional role of α5β1 integrin-fibronectin interaction [18]. These studies implicate a role for FNMA in potentially mediating cohesion and invasion in prostate cancer. Accordingly, we set out to determine whether prostate cancer cell lines of different invasive potentials also differed markedly in their capacity for FNMA and if so, whether this was connected to differences in cohesion.

The model we chose to use is the Dunning rat prostate cancer model [19]. This model is ideal for these studies since lines have been established that are differentially invasive and metastatic [20], and that also display differential adhesion to various ECM components, including fibronectin [21]. None of the lines used express E-cadherin on their surface [22]. We first set out to measure the cohesivity of aggregates of three cell lines; the none tumorigenic JHU-3, the tumorigenic but none-invasive AT-2, and the anaplastic, invasive, and metastatic cell line MAT-LyLu (MLL). Cohesion was measured by tissue surface tensiometry (TST). The biophysical concepts underlying TST have been previously described in detail [23,24]. This method entails the compression of spherical aggregates between parallel plates in a custom-built tensiometer chamber [25]. Compressions are conducted under physiological conditions and proceed until aggregates reach shape and force equilibrium, whereupon, aggregate geometry and the resistance force are measured. These measurements are then applied to the Young-Laplace equation [26], generating measurements of aggregate cohesion, otherwise expressible as tissue surface tension. We next assessed FNMA by the three lines. To establish a functional role for FNMA, we generated cell lines that express either wild-type α5 integrin, or a chimeric construct in which the cytoplasmic domain of α5 was switched to that of α2 integrin, an integrin that does not support FNMA [16]. We then explored effects on FNMA, aggregate compaction, cohesion, and invasion. We also treated MLL cells with AZD6244, a selective MEK-inhibitor previously demonstrated to promote FNMA and explored its' effect on aggregate cohesion, tumor cell detachment, and actin organization.

We showed that multi-cellular aggregates of the three Dunning lines exhibit different levels of cohesion that correlate inversely with their invasiveness. We also demonstrated a correlation between aggregate cohesion and FNMA. Moreover, we establish a functional role for FNMA in mediating tumor cell detachment by showing that restoring matrix assembly of invasive cells renders them significantly less invasive. This is the first demonstration that the fibronectin matrix can act as an invasion suppressor by effectively increasing the cohesion of 3D aggregates of prostate cancer cells.

## Methods

### Cell lines

Three well-characterized cell lines from the Dunning rat prostate cancer model [19,27] were used for all studies. JHU-3 cells were obtained from the American Type Culture Collection (ATCC, Manassas, VA). MAT-LyLu and AT-2 cells were a kind gift from Dr. William Isaacs (Johns Hopkins University). JHU-3 and MAT-LyLu (MLL) cells were maintained in RPMI-1640 medium (Invitrogen, Carlsbad, CA) supplemented with 10% fetal calf serum (FCS, Hyclone, Rockford, IL), 1% non-essential amino acids (NEAA, Invitrogen), 1% antibiotic/antimycotic (AA, Invitrogen), and 250 nM dexamethasone (Sigma, St. Louis, MO). AT-2 cells were maintained in DMEM supplemented with 10% FCS, 1% NEAA, and 1% AA mixture. Normal rat fibroblasts (Rat-2) were obtained from the ATCC and maintained in DMEM supplemented with 10% FCS and 1% AA.

### Treatment of MLL cells with the MEK inhibitor AZD6244

When required, MLL cells were plated at 60% confluence, allowed to adhere for 24 hours, then treated overnight with 1.5 μM of AZD6244, or with a corresponding volume of DMSO as a carrier control. Cells were then used as described below for generation of spheroids for measurement of aggregate cohesion by TST, for assessment of FNMA by immunofluorescence or immunoblot assay, and to perform 2D and 3D assays.

### Measurement of aggregate cohesion by tissue surface tensiometry

Detailed methods describing the procedure have been previously published [3,23,24] and are presented in "Additional file 1."

### Invasion assays

Assays were performed using both single cell suspensions and 3D aggregates. Cells were detached with 0.5 g/L Trypsin-0.2 g/L EDTA (TE, Invitrogen), counted using a BioRad TC10 automated cell counter, and resuspended at a concentration of 5 ×10^5 ^cells/ml in serum-free DMEM. 100 μl were plated into either BD Biocoat Matrigel transfilter invasion chambers or control inserts lacking a Matrigel barrier (BD Biosciences, Bedford, MA). Immediately after adding cells, 100 μl of serum-free medium was added to each chamber and these were then transferred into wells of a 24-well tissue culture plate containing 250 μl of 10x conditioned medium as a chemo-attractant. Chambers were incubated for 24 hours, whereupon cells on the top surface of the filter were scraped off using a cotton swab moistened with serum-free DMEM. Filters were then transferred to fresh wells containing 300 μl of a 1 μM solution of the fluorescent nuclear dye Syto-16 (Invitrogen, Carlsbad, CA). Images of fluorescent nuclei of cells that had traversed the membranes from four-10x fields for each insert were captured using a Nikon Eclipse TE 300 epifluorescence microscope connected to a CoolSnap ES digital camera. Image analysis was performed using ImageJ. The invasion index was calculated by dividing the number of invading cells (Matrigel filters) by the number of migrating cells (control inserts). For 3D invasion assays, cell suspensions were adjusted to a concentration of 1 × 10^6 ^cells/ml and 10 μl hanging drops were formed as described in Additional file 1. Aggregates ranging in size from 50-70 μm were placed into Matrigel invasion chambers (Becton-Dickinson, MA) containing serum-free medium and transferred into wells of a 24-well plate containing 250 μl of 10x-conditioned medium as a chemo-attractant. A total of nine aggregates from each cell line were placed, three aggregates per well, in nine invasion chambers. After 24 hours in culture, a cotton swab was used to remove the aggregates and non-invading cells from the top of the filter. Cells that had traversed the membrane were stained and quantified as described above.

### 3D growth rate assay

In order to determine whether AZD treatment could influence the growth rate of aggregates of MLL cells, thereby contributing to any observed differences in the number of cells counted on the underside of the membrane, hanging drops of MLL-DMSO and MLL-AZD6244 were generated and incubated for a period of time corresponding to that used for the invasion assays. Typically, batches of aggregates were incubated for 5-days at which time 10 aggregates from each batch were pooled and dissociated in trypisn-EDTA. The number of cells were counted for days 6-8. Linear regression analysis was then used to determine whether growth rates differed between DMSO and AZD-6244 treated cells.

### Assessment of fibronectin matrix assembly and actin organization by immunofluorescence (IF) microscopy

For assessment of FNMA, cells were plated into 24-well tissue culture plates at a density of 5 × 10^5 ^cell/ml in tissue culture medium containing 10% fibronectin-depleted FCS. Serum was depleted of fibronectin by incubation with Gelatin Sepharose 4B (GE Healthcare, Piscataway, NJ) as previously described [28]. Thirty-μg/ml of rat plasma fibronectin (Calbiochem, Los Angeles, CA) was added to each well and the plates were incubated for 24 hours under standard conditions. After 24 hours in culture, cells were washed twice with HBSS and blocked in CAS-Block buffer (Invitrogen, Carlsbad, CA) for 30 minutes. Fibronectin matrix was detected by incubating cells in anti-FN antibody (ab6584, Abcam, Cambridge, MA) for one hour at RT, and again after 3 washes with HBSS, in Alexafluor-568 or Alexafluor 488 secondary antibody (Invitrogen, Carlsbad, CA) for 30 minutes. After washing twice with HBSS, cells were counterstained with DAPI and imaged by epifluorescence microscopy. Images from the red or green and UV channels were captured and merged in IPLab imaging software. For assessment of actin organization, cells were washed in PBS, then fixed and permeabilized in 4% paraformaldahyde/0.1% Triton X-100 for 15 minutes at room temperature. After washing with PBS, cells were incubated in 1:40 rhodamine-phalloidin (Invitrogen, Carlsbad, CA):PBA and 1:1000 DAPI for 15 minutes, rinsed 2X in PBS, mounted in Fluorosave reagent (Calbiochem) and imaged as described above.

### Assessment of FNMA by differential solubilization assay

The assembly of high molecular weight FN multimers (HMWFM) was assessed using deoxycholic acid (DOC) differential solubilization as previously described [16,29]. Cells were lysed in a DOC lysis buffer (2% w/v sodium deoxycholate, 20 mM Tris-HCl pH 8.8, 2 mM PMSF, 2 mM EDTA, 2 mM iodoacetic acid, and 0.2 mM *N*-ethylmaleimide), passed through a 26-gauge needle, and centrifuged at 16,000 xg for 15 minutes at 4°C. The supernatant containing the DOC soluble fraction was transferred to a fresh tube. The pellet from the 15-minute spin, representing the DOC-insoluble fraction, was solubilized using SDS lysis buffer (1% SDS, 20 mM Tris-HCl, pH 8.8, 2 mM PMSF, 2 mM EDTA, 2 mM iodoacetic acid, and 0.2 mM *N*-ethylmaleimide). Protein fractions were separated by SDS-PAGE under reducing conditions. Protein was transferred to PVDF and blocked for 4 hours in 5% nonfat dry milk/TBST (Blotto). Blots were then incubated in anti-FN antibody (ab6584, Abcam, Cambridge, MA) at a concentration of 1:2,000 in Blotto at 4°C for 16 hours. After several washes in TBST, blots were probed with streptavidin-HRP (1:5,000 in Blotto) for 1 hour at room temperature, washed, and developed using enhanced chemiluminescence (Western Blot Detection System, GE Healthcare). Blots were also probed for Actin (A2066, Sigma, St. Louis, MO) to control for equal loading.

### Assessment of α5β1 cell surface integrin expression by flow cytometry

Cells were detached from near-confluent tissue culture plates with TE (Invitrogen, Carlsbad, CA), washed three times with ice-cold HBSS, and resuspended at a concentration of 1 × 10^7 ^cells/ml. One hundred μl aliquots, in duplicate, were deposited into 15-ml conical centrifuge tubes. Five μg/ml of anti-integrin antibody (α5/FnR mouse monoclonal P1D6, Calbiochem, Billerica, MA) was added to one of the duplicates and tubes were incubated on ice for 30 minutes with agitation. After two washes with HBSS, cells were re-suspended in a 1:100 dilution of Alexa-Fluor 488-conjugated goat-anti-mouse IgG (Invitrogen, Carlsbad, CA). After 30 minutes, cells were washed twice and analyzed using a Becton-Dickinson FacsCalibur flow cytometer and CellQuest software. Mean fluorescence intensity (MFI) values were normalized by subtracting the MFI of IgG-FITC controls from those of the α5β1 integrin-specific signal.

### Generation of Chimeric α5 integrin-expressing cells

MLL cells were transfected by electroporation with 30 μg of α5 cDNA constructs which encode the extracellular domain of α5 integrin and the cytoplasmic domain of either α5 (MLL-X5C5) or α2 integrin (MLL-X5C2) as described in [30]. Transfected cells were grown for 24 hours, and then selected in 800 μg/ml of G418 until resistant cells reached 40-50% confluence. Cells were detached with TE, washed three times with ice-cold HBSS, and incubated with an anti-human α5β1 integrin antibody (α5/FnR, Calbiochem) at 5 μg/ml on ice for 45 minutes. Cells were washed with cold HBSS and incubated on ice for an additional 45 minutes with an Alexafluor-488-conjugated goat-anti-mouse secondary antibody (Invitrogen). Cells expressing similar levels of α5 integrin were bulk-sorted by FACS (EPICS ALTRA, Beckman Coulter, FL), expanded, and maintained in 400 μg/ml of G418.

### Compaction assay

Ten-microliter hanging drops containing 25,000 cells each were incubated for 24 hours in complete medium or medium containing 50 μg/ml of the 70 kDa fibronectin fragment. Within this time frame, cells coalescing at the bottom of the hanging drops formed sheets. Images were captured, outlines were automatically traced, and the number of pixels within the outlines were quantified using IP Lab imaging software. Data points representing the mean and standard error for aggregate size in pixels were calculated from 10 hanging drops each of MLL, MLL-X5C2, and MLL-X5C5.

### Statistical analysis

The mean surface tensions, differences in invasion index, and compaction of the Dunning lines were compared by ANOVA and Tukey's Multiple comparisons test. Mean surface tension after the first and second compressions, and difference between initial applied force and surface tension were compared by Student's *t*-test. The relationship between surface tension and aggregate volume and the growth rate data were analyzed by linear regression.

## Results

### Tissue surface tension measurements of aggregates of dunning CaP cells

**Figure 1:**
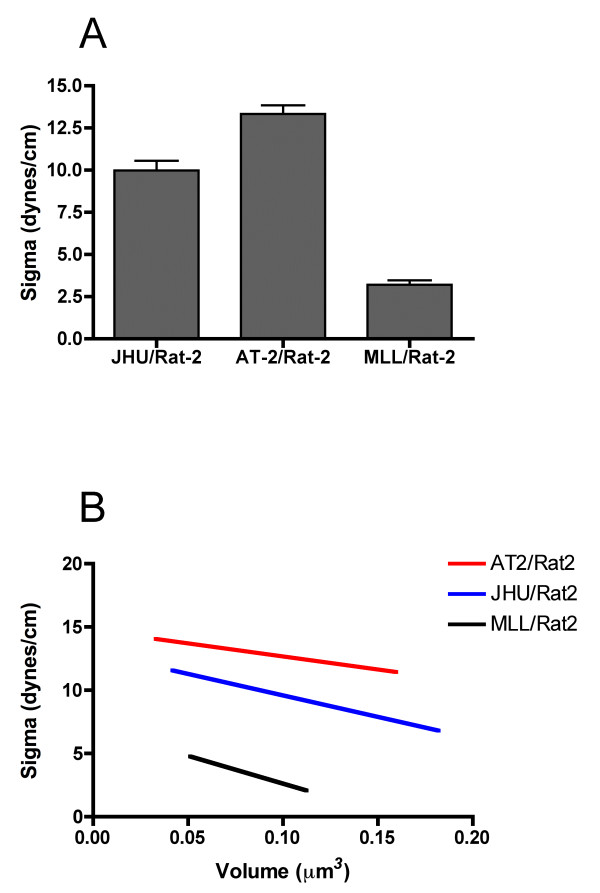
**TST measurements of Dunning rat CaP aggregates**. Comparison of aggregate cohesion by TST shows that aggregates composed of a 4:1 mixture of JHU-3: Rat-2 and AT-2: Rat-2 are significantly more cohesive than those of MLL: Rat-2 (ANOVA, *p *< 0.0001, Tukey's MCT, JHU-3:AT-2 *p *< 0.001, JHU-3-MLL *p *< 0.001, AT-2:MLL *p *< 0.001). The data set is comprised of n = 20 JHU-3, n = 21 AT-2, and n = 20 MLL aggregates, representing 40, 42, and 40 compressions, respectively (**A**). Aggregate cohesion is independent of volume. The relationship between σ and aggregate volume was assessed by linear regression analysis. For liquid-like aggregates, σ must be independent of volume. This was confirmed by showing that the correlation coefficient, R^2^, of regression lines for MLL, AT-2, and JHU-3 are 0.23, 0.06, and 0.07, respectively, indicating that σ is independent of volume (**B**).

TST measurements of aggregates of the Dunning lines reveal that JHU-3 and AT-2 are significantly more cohesive with surface tensions (σ) of 9.9 ± 0.6 and 13.1 ± 0.5 dynes/cm, respectively, than those of MLL with a σ of 3.2 ± 0.3 dynes/cm, as compared by ANOVA (*p *< 0.0001) and Tukey's MCT (Table 1 and Figure 1A). The TST measurements were validated by showing that (i) σ measured after the first compression (σ_1_) is not significantly different than that measured after a second, greater compression (σ_2_), (ii) the ratio of σ_2_/σ_1 _approaches 1, (iii) the ratio of the initial applied force at both compressions (F_2_/F_1_) is significantly greater than the ratio of σ_2_/σ_1 _(Table 2), and (iv) that σ is independent of aggregate volume (Figure 1B).

**Table 1:** Tissue surface tension measurements of Dunning prostate cancer lines

Line	σ_1 _(dynes/cm ± s.e.m.)	σ_2_(dynes/cm ± s.e.m.)	P σ_1 _vs σ_2_	σ_1,2_(dynes/cm ± s.e.m.)
JHU	10.2 ± 0.9	9.8 ± 0.8	0.66	9.9 ± 0.6

AT-2	12.5 ± 0.7	13.7 ± 0.7	0.23	13.1 ± 0.5

MLL	3.1 ± 0.3	3.3 ± 0.4	0.74	3.2 ± 0.3

MLL-X5C2	2.7 ± 0.3	2.9 ± 0.3	0.56	2.8 ± 0.2

MLL-X5C5	13.6 ± 1.9	13.2 ± 1.6	0.90	13.4 ± 1.1

Rat 2	19.7 ± 1.9	22.8 ± 2.2	> 0.05	21.1 ± 1.4

**Table 2:** Confirmation of aggregate liquidity

Line	σ_2_/σ_1_	F_2_/F_1_	$\frac{F_{2}/F_{1}}{\sigma_{2}/\sigma_{1}}$	P
JHU	0.95 ± 0.03	1.43 ± 0.02	1.54 ± 0.07	< 0.05

AT-2	1.11 ± 0.02	1.43 ± 0.02	1.29 ± 0.03	< 0.05

MLL	1.00 ± 0.04	1.45 ± 0.03	1.45 ± 0.07	< 0.05

MLL-X5C2	1.13 ± 0.04	1.52 ± 0.07	1.39 ± 0.11	< 0.05

MLL-X5C5	1.01 ± 0.08	1.45 ± 0.03	1.57 ± 0.07	< 0.05

Rat 2	1.14 ± 0.05	1.60 ± 0.06	1.40 ± 0.15	< 0.05

### Invasion index is inversely proportional to aggregate surface tension

**Figure 2:**
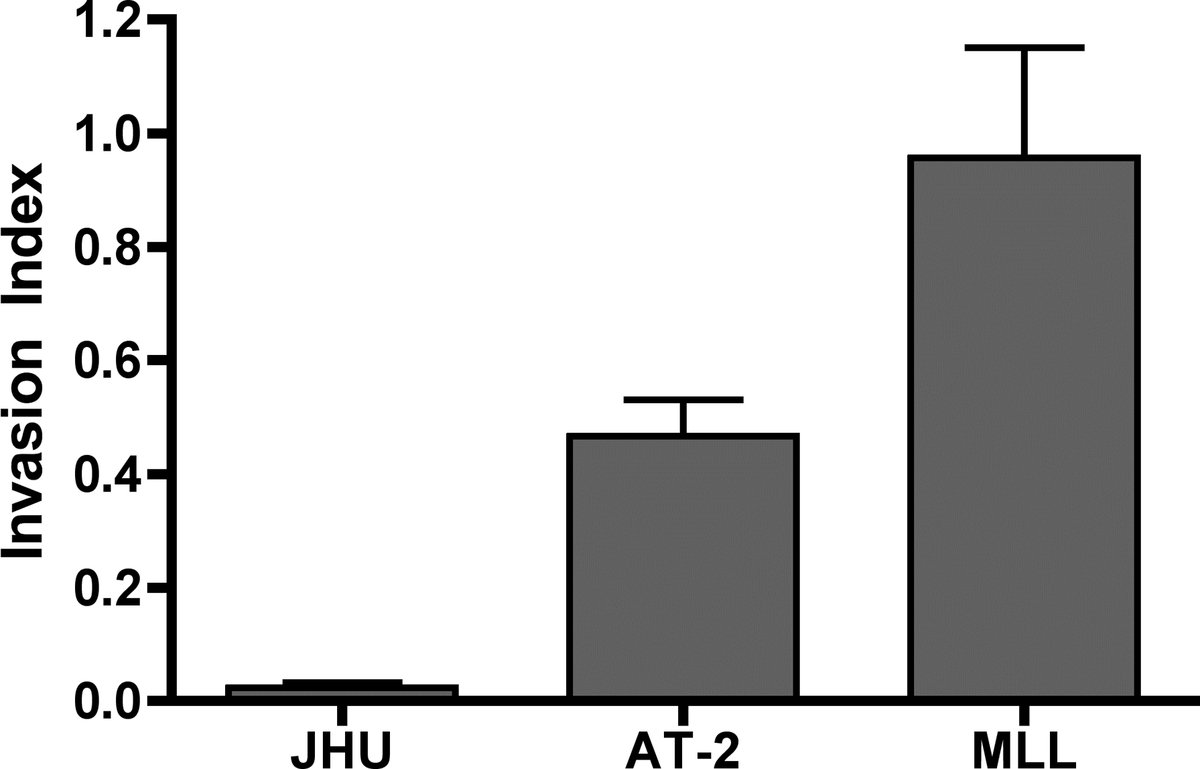
**Invasion index of Dunning rat CaP cells**. MLL cells are significantly more invasive than either AT-2 or JHU when invasion indices are compared by ANOVA (*p *< 0.0003). Tukey's MCT detected a significant difference in invasion index between JHU-3 and AT-2 cells (*p *< 0.05), JHU-3 and MLL cells (*p *< 0.001), and AT-2 and MLL cells (*p *< 0.05).

As can be seen in Figure 2, JHU-3 cells are, for all practical purposes, non-invasive, with an index of 0.023 ± 0.008, whereas AT-2 appear to be somewhat more invasive with an index of 0.47 ± 0.06. MLL cells are the most invasive, with an index of 0.94 ± 0.18. In general, invasive index appears to be inversely proportional to surface tension, with MLL cells being the least cohesive and most invasive, whereas JHU-3 and AT-2 cells tend to be more cohesive and less invasive (ANOVA, *p *= 0.0003).

### Fibronectin matrix assembly by dunning CaP cells

**Figure 3:**
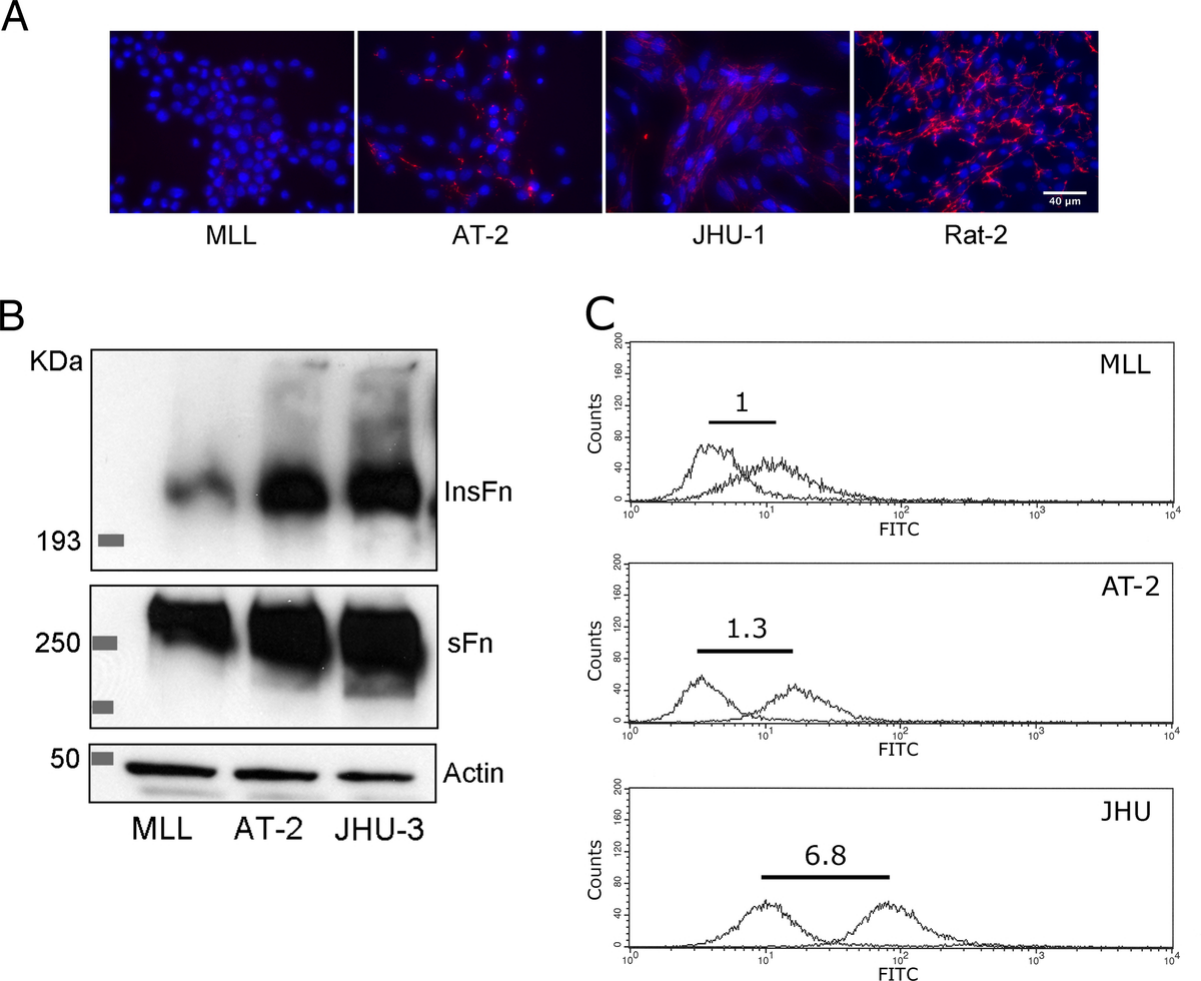
**FNMA by Dunning CaP cells**. Immunofluorescence analysis shows that MLL cells cannot assemble a matrix (**A**), whereas AT-2 (**B**) and JHU-3 (**C**) cells can do so to some extent. Neither of the CaP cells, however, can assemble a matrix to the same extent as Rat-2 fibroblasts (**D**), used here as a positive control. DAPI staining was used to demonstrate equal cell density. Scale bar represents 40 micrometers (**A**). Biochemical analysis of FNMA by Dunning cells. The assembly of high molecular weight fibronectin multimers (HMWFM) by MLL, AT-2, and JHU-3 cells was assessed using DOC differential solubilization and immunoblot analysis. Actin in the soluble fraction was used as a loading control. The presence of HMWFM is higher in AT-2 and JHU-3 cells than in MLL (**B**). α5β1 surface expression by Dunning CaP cells. Analysis of normalized MFI for α5β1 integrin expression by the Dunning lines shows that JHU-3 cells express approximately 5-fold more receptors than AT-2 and 7-fold more than MLL. MFI was normalized by subtracting the MFI of the IgG-FITC control histogram from that of the α5β1-integrin specific histogram and expressing net MFI of each cell line relative to that of MLL (**C**).

FNMA has been previously shown to mediate cell-cell cohesion in 3D aggregates [30]. Accordingly, these three cell lines were assessed for their ability to assemble fibronectin into a matrix. As can be seen in Figure 3A, MLL cells lack the capacity for FNMA, whereas AT-2 and JHU-3 tend to assemble a richer fibronectin matrix. FNMA was also assessed using a differential solubilization assay and immunoblot analysis. Figure 3B confirms that the amount of HMWFM detected by immunoblot analysis was significantly less in MLL than in AT-2 and JHU-3 cells. One possible explanation for differential capacity for FNMA may be associated with different levels of α5β1 integrin receptor expression. Accordingly, we used flow cytometry to specifically compare cell surface receptor expression by the three Dunning lines. Figure 3C shows that MLL cells express approximately 7-fold fewer α5β1 integrin molecules on their surface than JHU cells. We thus asked whether increasing expression of α5β1 integrin by MLL cells would result in increased capacity for FNMA and higher aggregate cohesion.

### Chimeric α5-integrin expression by MLL cells

**Figure 4:**
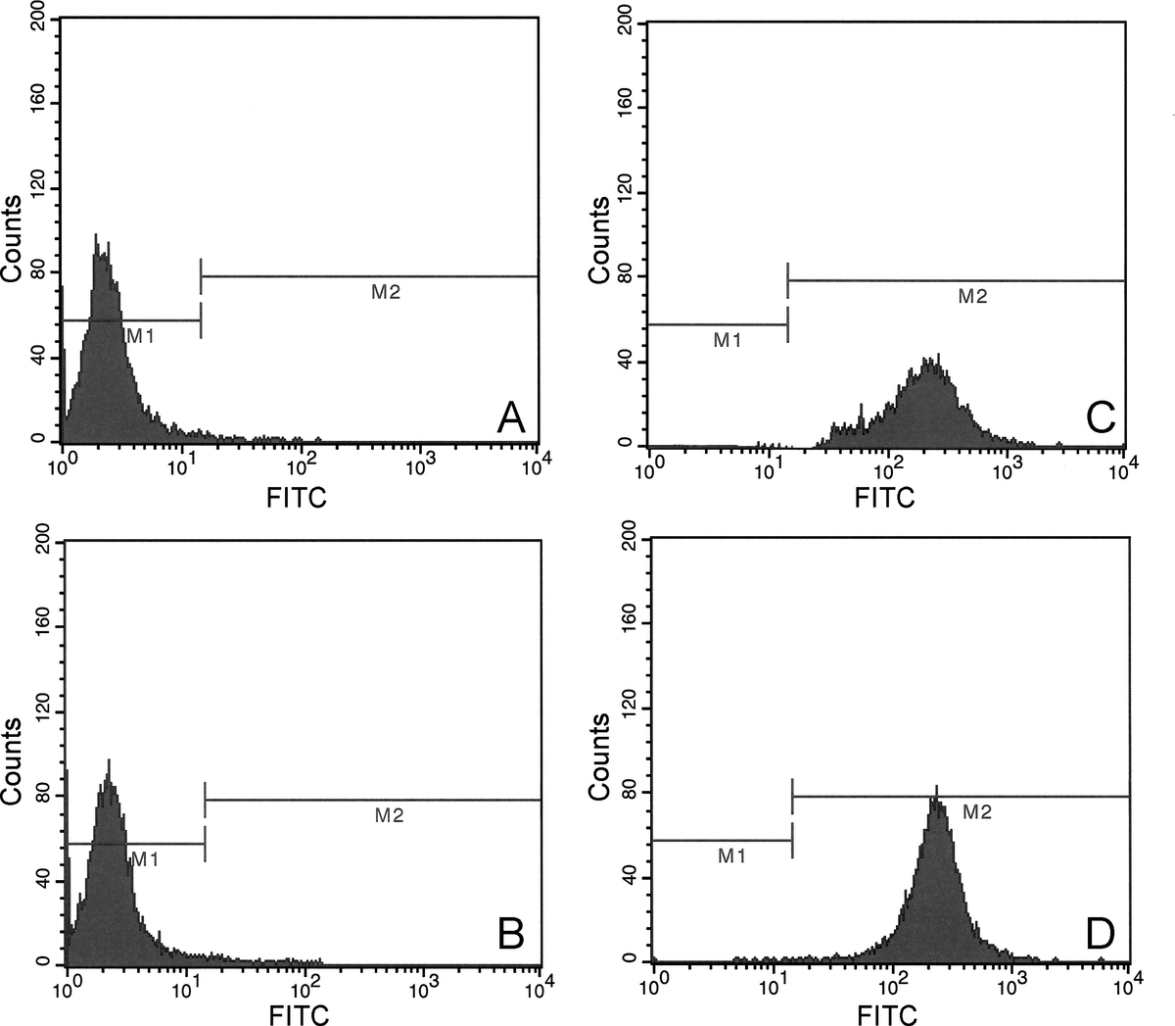
**Cell surface expression of chimeric integrins by MLL cells**. Flow cytometry analysis reveals that MLL cells do not react with anti-human α5 integrin antibody PD16 (**B**). In contrast, PD16 is able to detect expression of the α5 extracellular domain of X5C2 (**C**) and X5C5 (**D**), as denoted by a significant increase in mean peak channel fluorescence relative to the IgG-FITC controls (**A**).

We transfected MLL cells with cDNA encoding for expression of the extracellular domain of α5 integrin and the cytoplasmic domains of either α5 integrin (X5C5) or α2 integrin (X5C2). Previous studies have shown that whereas X5C5 can promote the assembly of a rich fibronectin matrix, expression of X5C2 gives rise to short, punctate clusters [30]. We then used flow cytometry to generate cell lines that were matched in their levels of α5-integrin expression. We used unstained MLL cells to establish baseline endogenous fluorescence (Figure 4A), and an antibody against the extracellular domain of human α5 integrin to detect the transfected protein. Figure 4B shows that the antibody does not recognize rat α5 integrin, whereas it can readily detect the transfected X5C2 (4 C) and X5C5 (4D) extracellular domains. The levels of integrin expression by MLL-X5C2 and MLL-X5C5 appears to be similar as denoted by significant overlap of the histograms (Figure 4). To quantify the data, we ran the experiment 5 times and generated values for mean fluorescence intensity (MFI). MFI for MLL-X5C2 and MLL-X5C5 were 217.8 ± 57.2 and 232.8 ± 88.9 units, respectively. A Student's *t*-test was used to compare the means, and showed that α5 expression levels are not statistically different (*p *= 0.8447), confirming matched levels of expression.

### Integrin expression promotes FNMA by MLL cells

**Figure 5:**
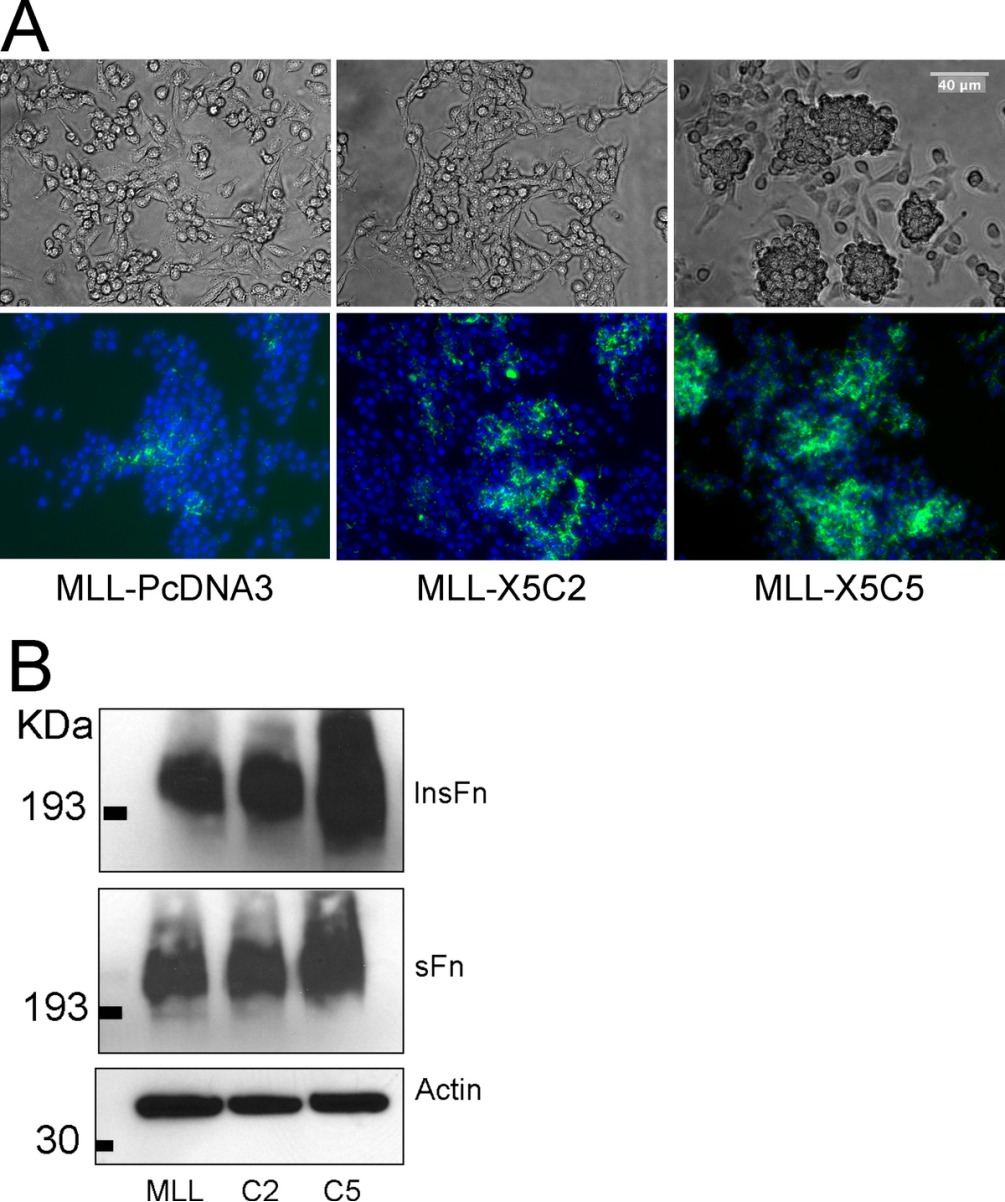
**Expression of chimeric integrins alters cell-cell interactions and capacity for FNMA**. Phase contrast and immunofluorescence images of MLL, MLL-X5C2, and MLL-X5C5 cells show that, whereas untransfected MLL cells tend to form loose colonies with very low capacity for FNMA, MLL-X5C2 cells form tighter colonies and acquire a limited capacity for matrix assembly. In contrast, MLL-X5C5 cells tend to aggregate into spheroids that lift off the surface of the culture dish. These cells also appear to assemble a rich fibronectin matrix. DAPI staining was used to demonstrate equal cell density (**A**). Biochemical analysis of FNMA by chimeric integrin-expressing cells. The assembly of high molecular weight fibronectin multimers (HMWFM) by MLL, MLL-X5C2, and MLL-X5C5 cells was assessed using DOC differential solubilization assay. DOC-soluble and insoluble fractions were separated by SDS-PAGE and analyzed by immunoblotting using an anti-fibronectin antibody. Actin in the soluble fraction was used as a loading control. The presence of HMWFM is highest in MLL-X5C5 cells (**B**).

We assessed the effect of integrin expression on FNMA. As can be seen in Figure 5A, when plated onto tissue culture plastic, MLL cells tend to form loose colonies that do not appear to assemble a matrix. MLL-X5C2 cells appear to form tighter colonies that contain areas in which matrix assembles in short, punctate fibrils. MLL-X5C5 cells tend to lift off the plate to form spheres that are loosely attached to the dish and assemble a denser fibronectin matrix. This was confirmed by biochemical assessment of FNMA using a differential solubilization assay and immunoblot analysis. Figure 5B shows that only MLL-X5C5 cells (lane C5) express large amounts of HMWFM.

### MLL cells expressing different Chimeric integrins demonstrate different compaction behavior

**Figure 6:**
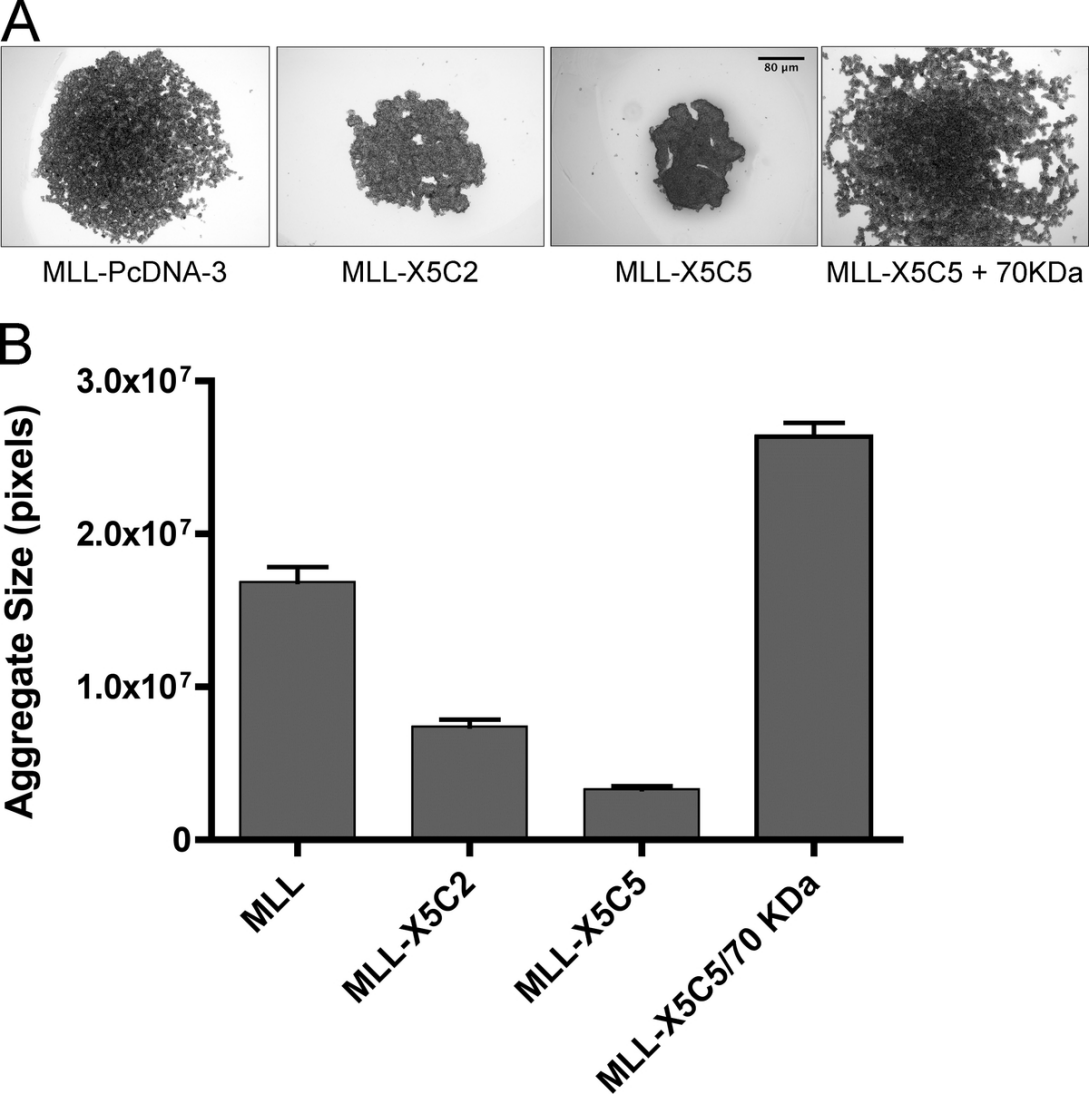
**Compaction of chimeric integrin-expressing cells**. Hanging drop cultures of parent MLL and chimeric integrin-expressing cells show that expression of the different chimeric integrins can markedly influence aggregate compaction. When placed in hanging drop culture, parent MLL cells tend to generate large cellular sheets. Expression of X5C2 gives rise to more compact sheets, whereas expression of X5C5 results in even more compact aggregates. Compaction of MLL-X5C5 cells can be blocked by incubation in 50 μg/ml 70 kDa fibronectin fragment (**A**). Quantification of aggregate compaction. Compaction was quantified and statistically analyzed by ANOVA and Tukey's MCT. ANOVA detected significant size difference within the data set (*p *< 0.0001). Tukey's MCT confirmed that MLL-X5C5 are more compact than MLL (*p *< 0.001) and MLL-X5C2 (*p *< 0.01). Moreover, incubation of MLL-X5C5 with 50 μg/ml 70 kDa fragment of fibronectin resulted in aggregates that were on average larger than MLL-X5C5 (*p *< 0.001) and MLL (*p *< 0.001) (**B**).

Figure 6A shows that MLL cells, when placed in hanging drop culture, tend to form loose sheets, whereas cells expressing X5C2 compact to some degree, but much less so than MLL-X5C5 cells, which tend to form much more compact aggregates. This compaction can be blocked by addition of 50 μg/ml of the 70 kDa fragment of fibronectin. We quantified compaction as a function of the expressed integrin. Figure 6B shows that MLL cells form cell sheets that are significantly larger than either MLL-X5C2 or MLL-X5C5 cells (ANOVA, *p *< 0.0001, Tukey's MCT, *p *< 0.001). Moreover, aggregates of MLL-X5C5 are significantly more compact than those of MLL-X5C2 (Tukey's MCT, *p *< 0.01). MLL cells incubated with 50 μg/ml of the 70 kDa fragment were, on average, larger than those generated by untransfected MLL cells (Tukey's MCT, *p *< 0.001), suggesting that the 70 kDa fragment of fibronectin interfered with some endogenous residual capacity for FNMA by MLL cells.

### Integrin expression specifies aggregate cohesion

**Figure 7:**
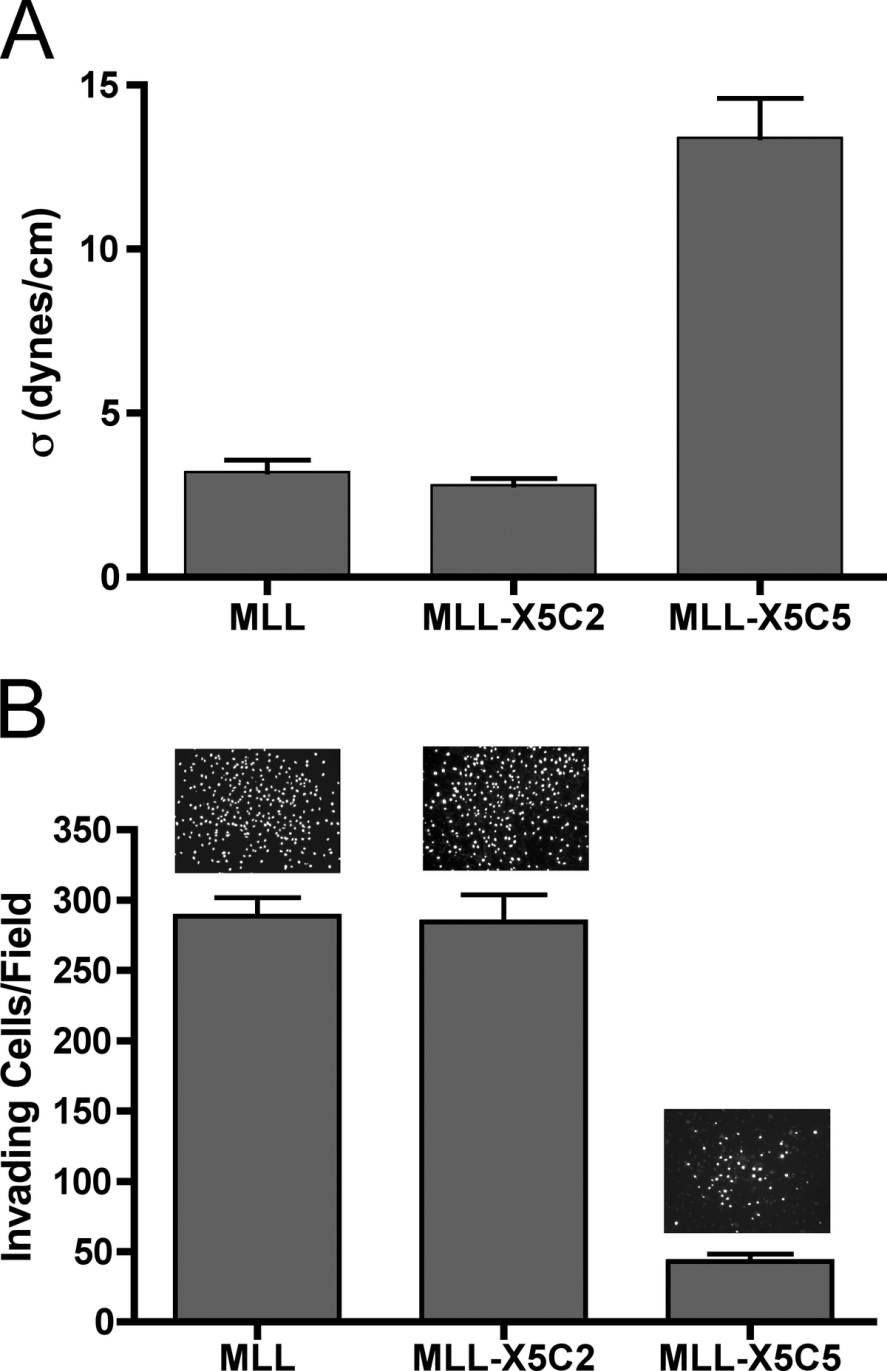
**TST measurements of chimeric integrin-expressing aggregates of MLL cells**. Comparison of aggregate cohesion by ANOVA and Tukey's MCT shows that aggregates of MLL-X5C5 cells are significantly more cohesive than those of the MLL parent line (*p *< 0.001) and MLL-X5C2 (*p *< 0.001). No difference in cohesion was detected between MLL and MLL-X5C2 (*p *> 0.05) (**A**). In vitro invasion of chimeric integrin-expressing MLL cells. The number of cells able to detach from n = 10 aggregates composed of MLL, MLL-X5C2, and MLL-X5C5, and to invade through a Matrigel filter was compared. Whereas no difference in invasiveness could be observed between MLL and MLL-X5C2 (*p *> 0.05), MLL-X5C5 cells were significantly less invasive than aggregates of either parent MLL (*p *< 0.001) or MLL-X5C2 (*p *< 0.001) (**B**). Representative images of invading cells are included as insets.

TST measurements reveal that aggregates of MLL-X5C5 are significantly more cohesive than those that express the X5C2 chimeric integrin. Table 1 and Figure 7A show that aggregates of MLL-X5C5 have a mean surface tension (σ) of 13.2 ± 1.6 dynes/cm, significantly higher than those of MLL and MLL-X5C2 that have a σ of 3.3 ± 0.4 and 2.9 ± 0.3 dynes/cm, respectively, as compared by ANOVA and Tukey's MCT. The TST measurements were validated as described above (Table 2).

### Integrin expression by MLL cells inhibits invasion

We assessed whether integrin expression could influence the ability of MLL cells to escape a 3D spheroid and invade through an ECM. We performed Matrigel transfilter invasion assays by depositing aggregates of MLL, MLL-X5C2 or MLL-X5C5 in the upper chamber and counted the number of cells that invaded into the lower surface of the filter. Figure 7B shows that aggregates of MLL and MLL-X5C2 were similarly invasive (Tukey's MCT, *p *> 0.05), and significantly more so than aggregates composed of MLL-X5C5 cells (Tukey's MCT, *p *< 0.001).

### The MEK inhibitor AZD6244 promotes FNMA, increases aggregate cohesion, and decreases tumor cell detachment

**Figure 8:**
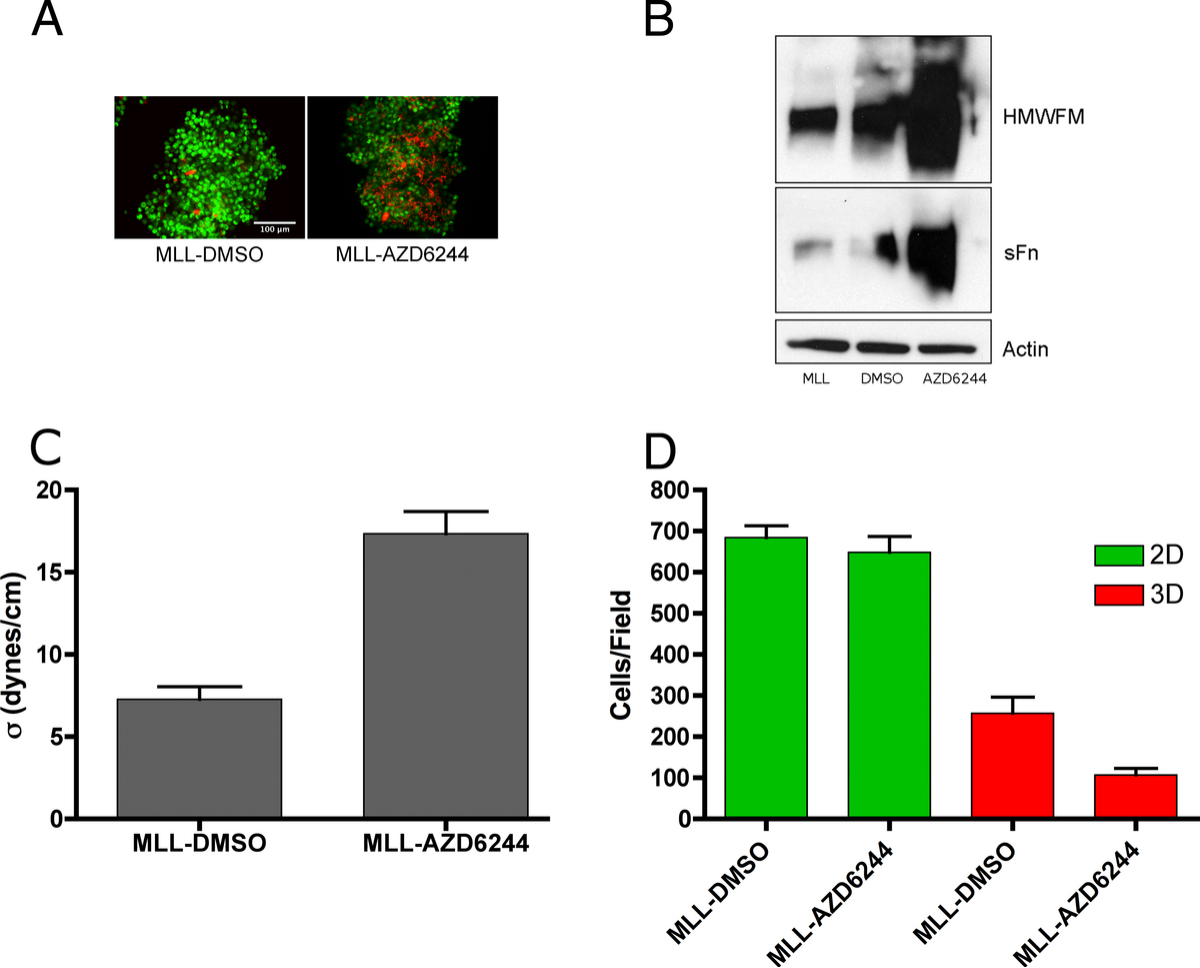
**AZD6244 promotes FNMA and decreases tumor cell detachment**. MLL cells treated with DMSO as carrier control fail to assemble a fibronectin matrix, whereas MLL cells treated overnight with 1.5 μM AZD6244 assemble a matrix as revealed by immunofluorescnce assay (**A**). Matrix assembly was confirmed by differential solubilization assay and immunoblot analysis. The assembly of high molecular weight fibronectin multimers (HMWFM) is only evident in AZD62244-treated MLL cells. Actin in the soluble fraction was used as a loading control (**B**). AZD6244 treatment also resulted in increased aggregate cohesion as measured by TST. Treated aggregates were found to be significantly more cohesive than aggregates treated with the DMSO carrier (Student *t*-test, *p *< 0.0001) (**C**). The migration of DMSO and AZD6244-treated MLL cells was not significantly different when cells were sparsely plated into Boyden chambers containing control filters (Student *t*-test, *p *= 0.4604). However, when migration assays were conducted using 3D spheroids of DMSO and AZD6244-treated cells, detachment of cells from the aggregate was shown to be significantly delayed by treatment with the MEK inhibitor (Student *t*-test, *p *< 0.0008) (**D**).

Various agents have been previously demonstrated to promote FNMA by cells deficient in this capacity, including the MEK inhibitor PD98059. Here, we treated MLL cells with AZD6244, a MEK inhibitor currently under investigation in clinical trials of melanoma. Treatment with AZD6244 clearly resulted in an increased capacity for FNMA when assessed by immunofluorescence (Figure 8A). This was confirmed by immunoblot analysis, which revealed that treatment with AZD6244 resulted in a marked increase in high molecular weight fibronectin multimers (Figure 8B). Restoring capacity for FNMA also gave rise in a marked increase in aggregate cohesion as measured by TST. The cohesion of aggregates composed of AZD6244-treated cells was found to be 17.3 ± 1.4 dynes/cm, whereas the cohesion of aggregates composed of untreated cells was significantly lower, at 7.3 ± 0.8 dynes/cm (Student's *t*-test, *p *< 0.0001, 8 C). When treated and control cells were sparsely plated (2D) into Boyden chambers containing uncoated filters, the number of cells migrating through the 8 μm pores were not significantly different from those treated with the DMSO carrier (Student *t*-test, *p *= 0.4604). However, when 3D aggregates were placed onto uncoated filters, the number of cells that were able to detach from the aggregate and migrate through the 8 μm pores was significantly lower for AZD6244-treated aggregates than for those treated with DMSO (Students *t*-test, *p *= 0.0008) (Figure 8D). To determine whether AZD6244 could potentially alter growth rate, as this could influence cell counts, we generated treated and control aggregates and measured growth rate of the 3D spheroids. Regression analysis revealed that the deviation from zero slope was not significant for either DMSO-treated (*p *= 0.9575) or AZD6244-treated cells (*p *= 0.9108). The r^2 ^values for the regression lines for DMSO and AZD6244-treated cells were 0.0002273 and 0.001319, respectively, indicating very little to no-growth within the time-frame of when aggregates were used for invasion or migration assays. No difference in the slopes was detected (*p *= 0.9365) (Additional file 1).

### AZD6244 activates α5β1 integrin

**Figure 9:**
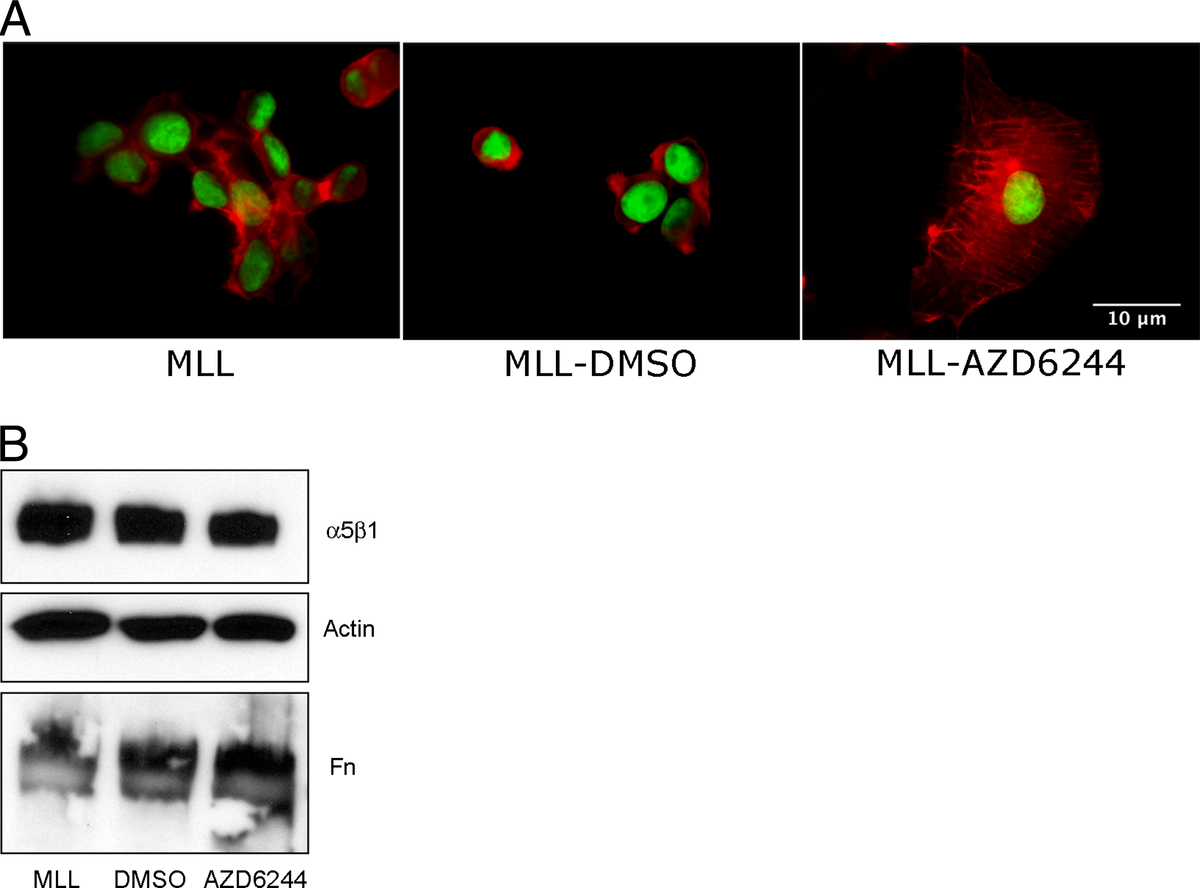
**AZD6244 treatment effects actin organization and reduces invasion**. Drug treatment results in a marked re-organization of the actin cytoskeleton. Here, MLL cells were sparsely plated and actin staining was performed using rhodamine-phalloidin and DAPI as a counterstain for nuclei. Actin organization changed from predominantly cortical for untreated and DMSO-treated cells to arrangement into stress fibers in response to the AZD6244 (**A**). This was accompanied by a change in cell shape from predominantly spherical and loosely adherent to flatter cells that appeared to adhere more strongly to the substrate. Immunoblot analysis revealed no change in either α5β1 integrin expression or in fibronectin secretion (**B**).

Apart from effects of AZD6244 on aggregate cohesion and cell detachment, we also asked whether treatment could also influence integrin activation. Figure 9A demonstrates that AZD6244 has a marked effect on actin organization and subsequently, on cell shape. Whereas untreated and DMSO-treated cells tend to remain somewhat spherical and were loosely adherent to the tissue culture plate, AZD6244 treatment resulted in marked cell spreading and flatter cells. This change in cell shape was accompanied by the reorganization of the actin cytoskeleton from predominantly cortical into stress fibers. Interestingly, this does not seem to be associated with an overt increase in expression of α5β1 integrin or in the secretion of fibronectin, both of which could also influence cell attachment (B), suggesting rather, that integrin function was somehow enhanced.

## Discussion

Various cancer cell lines have been previously demonstrated to generate spheroids when placed in hanging drop culture, including the lung cancer cell line Lewis Lung Carcinoma [1], human fibrosarcoma HT-1080 [31], cells of the glioblastoma U87-MG series [3,32], and breast cancer MCF-7 cells [33]. For these lines, aggregates either spontaneously formed spheroids or were induced to do so by embedding cells in extracellular matrix. Two of the three prostate cancer cell lines used in this study, AT-2 and JHU-3, spontaneously formed spheroids when placed in hanging drop culture. However, in order to generate sufficiently large spheroids for measurement of aggregate cohesion, it was necessary to admix normal rat fibroblasts with the MLL cells at a ratio of 1:4. The inclusion of fibroblasts provided sufficient motility within the aggregate to elicit the shape change required for aggregates to become spherical. Accordingly, we also admixed fibroblasts with AT-2 or JHU-3 cells so as to be able to compare aggregate cohesion between lines. We demonstrated an inverse relationship between aggregate cohesion and invasive index. We also showed that aggregate cohesion is independent of size and of the applied force [23,24], confirming that the TST measurements reflect real differences in cohesion between the three cell lines.

We also established a correlation between aggregate cohesion and capacity for FNMA. Earlier studies have ascribed a role for FNMA as a mediator of strong tissue cohesion in various cell lines including Chinese Hamster Ovary (CHO) cells [15,16], and cells derived from glioblastoma tumors [3]. This is the first demonstration that prostate cancer cells can vary in their ability to assemble a fibronectin matrix and that this correlates with aggregate cohesion, a property previously demonstrated to significantly influence cell detachment [3], aggregate spreading onto a substrate [34], and invasive capacity [1,2,32].

Alpha2Beta1 integrin, the receptor for collagen and other matrix molecules, is significantly down-regulated in poorly differentiated breast cancer [35], and has been demonstrated to suppress metastasis in mouse and human models of breast and prostate cancer [36]. We found a similar pattern of expression for α5β1; aggressive MLL cells expressing approximately 7-fold fewer receptors on their surface than JHU-3 cells. This could explain why MLL cells are deficient in their capacity to assemble a fibronectin matrix. Accordingly, we transfected MLL cells with α5 cDNA and bulk-selected a population of cells of increased α5β1 expression (MLL-X5C5). This resulted in increased FNMA, increased aggregate compaction, higher cohesion, and reduced invasive capacity. Compaction and cohesion could be blocked by incubation of MLL-X5C5 cells with the 70 kDa fragment of fibronectin, a fragment previously demonstrated to interfere with FNMA [37]. Accordingly, it was not possible to perform 3D invasion assays in the presence of the fragment in order to determine whether blocking matrix assembly results in rescue of the invasive phenotype. However, transfection of MLL cells with a chimeric integrin construct in which the cytoplasmic domain of α5 integrin was switched to that of α2 integrin (MLL-X5C2) did not increase aggregate cohesion or decrease invasion. This chimeric construct does not promote FNMA, rather, the fibronectin becomes localized in punctata and fibers do not extend between cells [30]. This further confirms that an intact matrix, assembled into fibers that extend between cells, is necessary to generate the force required to increase aggregate cohesion and discourage detachment of tumor cells and their subsequent invasion.

We propose that loss of the fibronectin matrix can promote invasion by facilitating the detachment of cancer cells from the tumor mass. Accordingly, loss of α5β1 expression or function represents a possible early mechanism whereby cells can proceed further down the metastatic pathway. Once cells have detached, they become free to move and undergo intravasation. Interestingly, loss of α2β1 integrin is associated with increased intravasation of breast cancer cells [36]. Since α2β1 is a receptor for collagen and other matrix molecules, it is also possible that loss of this receptor can also give rise to a decrease in tumor cohesion through decreased integrin-collagen (or other ECM) interactions in a similar fashion as the cohesion mediated through the interaction of α5β1 integrin and fibronectin [15]. Therefore, the combined loss of α5β1 and α2β1 could, in principle, markedly promote metastasis by controlling two key steps in the metastatic cascade: cell detachment and intravasation. The studies described above focus on integrin heterodimers that tend to be down-regulated in more aggressive cancers. Other integrin heterodimers have been shown to be over-expressed in aggressive tumors. AlphavBeta3 [38] and αvβ5 [39], for example, are currently being explored in clinical trials as potential targets of integrin antagonists [40]. Our study suggests that it may also be possible to reduce invasion and metastasis by developing integrin agonists that could act to reactivate integrin expression or function. This has already been demonstrated for glioblastoma cells, where reactivating FNMA by dexamethasone, the MEK-inhibitor PD98059, or the benzoquinone ansamycin antibiotic Geldanamycin, led to a significant increase in aggregate cohesion and reduced aggregate dispersal velocity [3]. More potent and FDA-approved MEK inhibitors, such as AZD6244, are currently being used in clinical trials for melanoma. Here we show that treatment of MLL cells with AZD6244 resulted in restoration of FNMA by MLL cells and that this manifested in a marked increase in tumor aggregate cohesion. Interestingly, drug treatment did not result in differences in the ability of single cells to migrate through an 8 μm filter, but rather, reduced the ability of tumor cells to detach from the 3D mass.

In conventional 2D cell culture, AZD6244 treatment of MLL cells resulted in a marked reorganization of the actin cytoskeleton and enhanced adhesion to the substrate, processes indicative of integrin activation. A recent report in melanoma cells also showed that AZD6244 induced actin reorganization and promoted integrin-mediated adhesion to substrate [41]. Integrin activation and cytoskeletal interaction are essential for the assembly of a fibronectin matrix [42]. Extended to 3D culture, increased affinity of integrin receptors for substrate could also contribute to the overall increase in aggregate cohesion observed here. On the one hand AZD6244 appears to promote aggregate cohesion, while on the other hand, it also appears to increase affinity of integrins for substrate and could, in principle also promote migration of cells away from the aggregate. These two opposing forces are physically interdependent. A previous study established an interplay between cell-cell and cell-substratum adhesion in mediating aggregate spreading [34] and it is likely that a similar relationship exists for aggregates of MLL cells. A shift in the balance favoring FNMA-mediated cell-cell cohesion is likely the case here.

## Conclusions

Collectively, the data suggest that an increase in tumor cohesion, mediated by restoration of FNMA, can act to suppress tumor cell detachment, and that it may be possible to pharmacologically impact an early step in the metastatic cascade of prostate cancer.

## Competing interests

The authors declare that they have no competing interests.

## Authors' contributions

DJ carried out the matrix assembly experiments, transfection of the MLL cells, flow cytometry, and compaction assays. IE performed the invasion, migration, and growth rate assays; CB performed the tensiometry experiments and analyzed the data; RAF conceived the experimental plan, analyzed the data, and drafted the manuscript. All authors read and approved the final manuscript.

## Financial Support

This study was supported by NCI grant R01-CA118755 to R.A.F.

## Pre-publication history

The pre-publication history for this paper can be accessed here:

http://www.biomedcentral.com/1471-2407/12/94/prepub

## Supplementary Material

Additional file 1 **Measurement of aggregate cohesion by tissue surface tensiometry **[43-45]. (DOC 275 kb). Click here for file
