# Supplementary material for: Fibronectin matrix-mediated cohesion suppresses invasion of prostate cancer cells
Source: BMC Cancer. 2012 Mar 20;12:94. doi: 10.1186/1471-2407-12-94 (PMC3359283; doi:10.1186/1471-2407-12-94)
Supplement: Additional file 1 — Measurement of aggregate cohesion by tissue surface tensiometry [43-45]. (DOC 275 kb). [file 1471-2407-12-94-S1.DOC]

**Supplemental information.**

**Measurement of aggregate cohesion by tissue surface tensiometry.**

**Aggregate formation.** Aggregates were prepared by the hanging drop method according to [43], with slight modification. Briefly, near-confluent monolayers of JHU-3, AT-2, MLL and RAT2 cells were detached from 10-cm tissue culture plates with trypsin/EDTA (TE). Complete medium was added to inhibit the trypsin and cells were centrifuged into a pellet then washed with PBS. Cells were resuspended in complete medium, counted using a BioRad TC10 automated cell counter (Hercules, CA), and adjusted to a concentration of 2.5 x10^6^ cells/ml. Mixtures of prostate cancer and Rat-2 fibroblast cells at an 4:1 ratio were then generated. Addition of fibroblasts facilitates spheroid formation. Ten-ml aliquots of JUH3:Rat2, AT-2:Rat2 or MLL:Rat2 were deposited on the underside of the lids of 10-cm tissue culture dishes. The bottom of the dish contained 5-ml of PBS and served to prevent evaporation of the drops by forming a hydration chamber. Hanging drops were created by inverting the lid over the hydration chamber. The drops were incubated at 37°C, 5% CO2, and 95% humidity for 24-48 hours allowing cells to coalesce and form spherical aggregates.

**Tissue surface tensiometry.**  Detailed methods describing TST have been previously reported [23, 24, 44]. A video article of the method can be found in [25]. Briefly, spherical aggregates ranging in size from 200-300 μ in diameter are loaded into the inner chamber of a custom-built tissue surface tensiometer (Fig. S1). The inner chamber contained pre-warmed, de-gassed CO_2_-independent medium (Gibco-BRL, NY) supplemented with 10% FCS and antibiotics. Single aggregates were compressed under physiological conditions between plates that were pre-coated with poly-2-hydroxyethylmethacrylate (poly-HEMA, Sigma, MO) to prevent adherence [45]. Aggregates were positioned on the lower compression plate (LCP), whose height could be adjusted. The upper compression plate (UCP), attached to a nickel-chromium wire, was then positioned above the aggregate and connected to a Cahn electro-balance. The weight of the UCP was zeroed to establish a pre-compression baseline. Compression was initiated by raising the LCP until the aggregate became compressed against the UCP. Adjusting the height of the LCP controlled different degrees of compression. Two parameters were measured: the force of resistance to compression, and aggregate geometry. The force with which the aggregate resisted compression was monitored by the electro-balance, which was connected to a chart recorder. Aggregate geometry was monitored through a 25x Nikon dissecting microscope equipped with a CCD video camera and connected to a Macintosh Power PC computer. Images of aggregates were captured, digitized and their geometries were analyzed using ImageJ (Bethesda, MD). Measurements of aggregate geometry and the force of resistance to the compressive force were then utilized in the Young-Laplace equation [26], producing numerical values of apparent tissue surface tension (σ) (Fig. S2 and Eqn. 1).

**Confirmation of aggregate liquidity.** A detailed analysis of aggregate liquidity has previously been reported in [17]. For the purpose of this study, 3 conditions were required in order to validate the surface tension measurements: 1) the calculated surface tension of an aggregate, when subjected to two successive compressions, the second (σ_2_) greater than the first (σ_1_), must remain constant. In such aggregates the ratio of σ_2_/σ_1_ will be equal to 1; 2) the ratio of σ_2_/σ_1_ will also be less than the ratio of the initial force applied at each successively greater compression (F_2_/F_1_); and 3) aggregate surface tension will also be independent of aggregate size. Only measurements in which surface tension is independent of the applied force and size were used to calculate average σ (true surface tension).


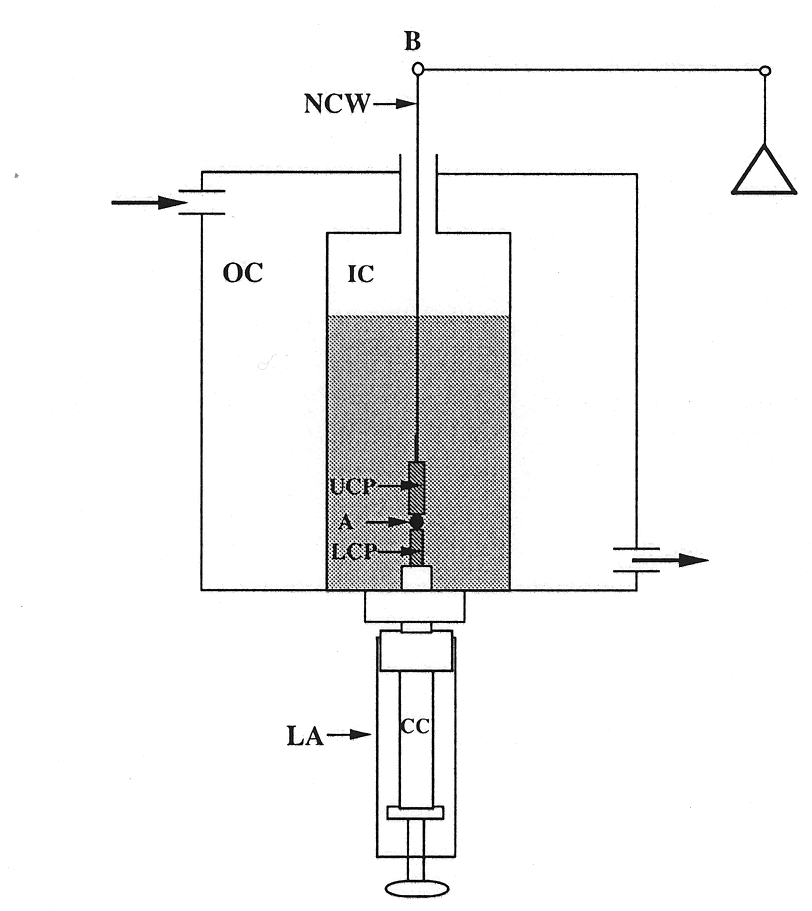


**Fig. S1. The tissue surface tensiometer.** The compression cell is composed of two chambers. The outer chamber (OC) is connected to a 37°C circulating water pump, and serves to regulate the temperature of the inner chamber (IC). The chambers are constructed of milled Delrin and contain quartz windows for visualization of the aggregate. The lower assembly (LA) screws into the base of the inner chamber and is used to 1) position the aggregate in the inner chamber; 2) seal the bottom of the inner chamber; 3) elevate the aggregate to initiate compression; and 4) control the distance between the parallel plates and hence the compression of the aggregate. The central core (CC) of the assembly is adjustable. The tip of the central core (the pedestal) is composed of smooth Teflon and acts as the lower compression plate (LCP). The upper compression plate (UCP) is a Teflon cylinder 15mm long that hangs from the balance arm (B) by a flame-straightened nickel-chromium wire (NCW). During the course of an experiment, the cell aggregate (A) is positioned on the lower plate and raised until it contacts the upper plate. The upper plate is connected to the balance arm (B). Compression of the aggregate causes displacement of the balance arm. The balance is a Cahn/Ventron model 2000 recording electrobalance, which operates on the null balance principle. The aggregate’s shape is monitored by visual observation through a 25X Nikon SMZ10A stereoscope coupled to an iMac computer. Images are captured and analyzed using ImageJ.


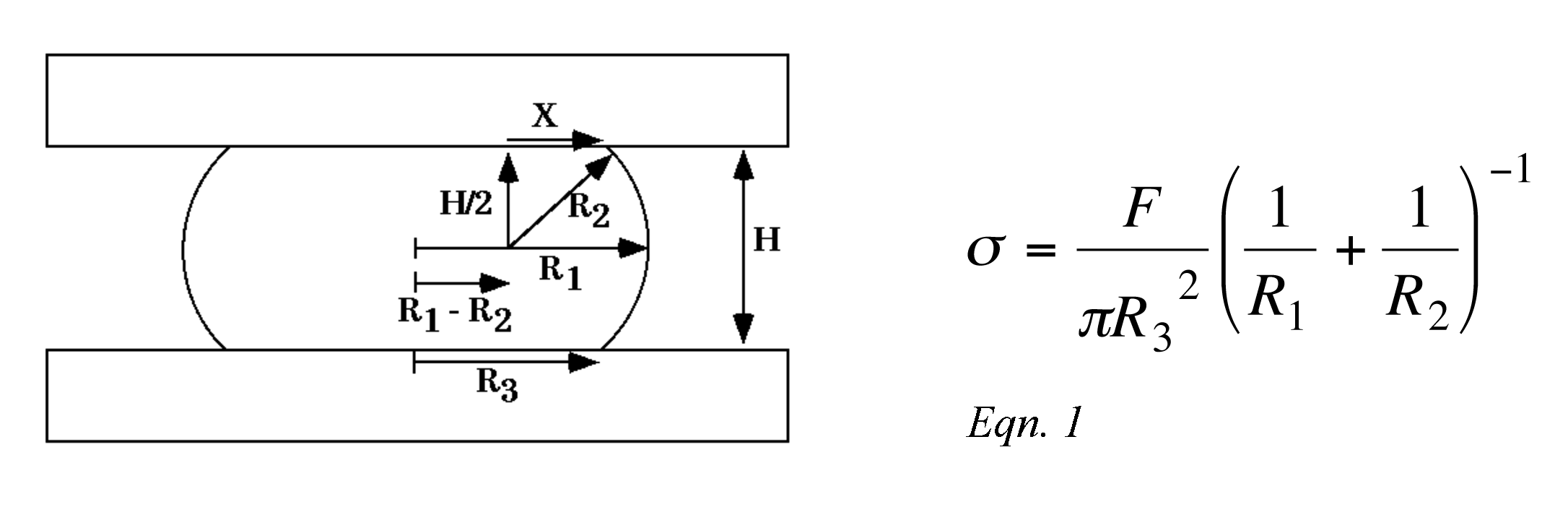


**Fig. S2. Calculation of aggregate surface tension.** At shape equilibrium, the cohesivity of an aggregate of cells compressed between parallel plates to which it does not adhere can be obtained from the Young-Laplace equation (Eqn. 1), where σ is cohesivity, F is the force acting to compress the aggregate, πr^2^_3_ is the area of the surface of the aggregate upon which force F is exerted, and R_2_ and R_3_ are, respectively, the radius of the equator of the compressed aggregate and the radius of an arc defining its surface profile normal to the compressing plates and extending between them. Measuring the compressive force and geometry at force and shape equilibrium and applying these measurements to the Young-Laplace equation generates numerical values of tissue surface tension.


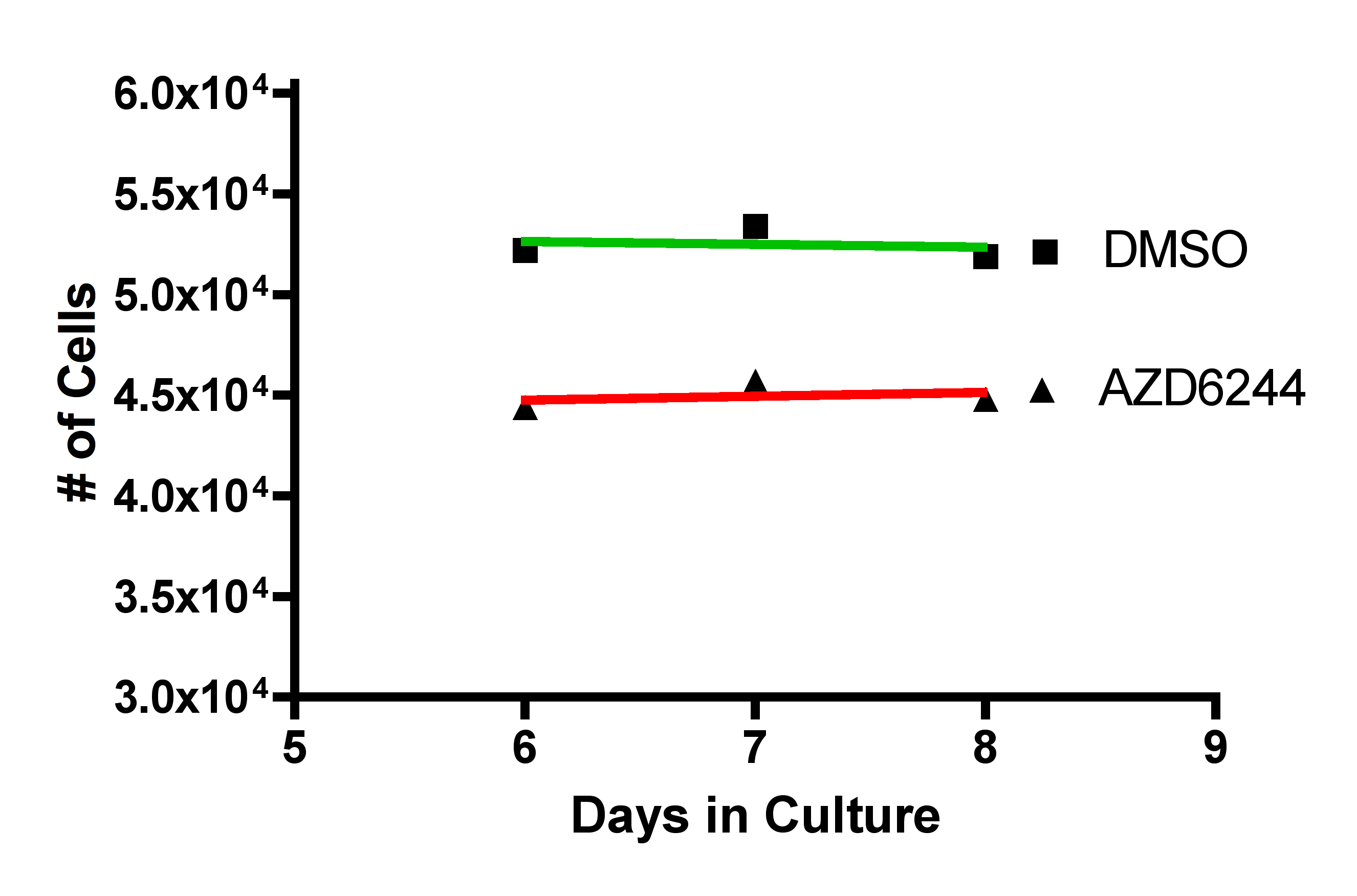


**Fig. S3. Growth rate of aggregates of DMSO and AZD6244-treated MLL cells.** Linear regression analysis of aggregate growth rate reveals that aggregates do not grow substantially within the time-frame in which they were used for migration assays.
